# Artificial Diseases and Their Treatment

**Published:** 1895-05

**Authors:** J. H. Allen

**Affiliations:** Logansport, Ind.


### ARTIFICIAL DISEASES AND THEIR TREATMENT.

J. H. Allen, M. D., Logansport, Ind.

I have read Dr. F. O. Pease's and Dr. Sawyer's articles in the *Medical Advance*, on the subject of artificial diseases and their treatment, with much interest. It is a subject that has interested me for years, and I wish to add my experience as further demonstration of the truth in this very important department of therapeutics.

For years I have been reporting cases through our journals in which the patient was either cured by antidoting the bad effects of crude drugs (artificial diseases) by a high potency of the same thing that produced it in the first place, or it cleared up the case to that point where the proper simillimum could be found for the natural disease, which was a thing impossible until I first removed the artificial disease.

Dr. E. W. Sawyer was the first one to call my attention to the fact that it was a law—the law of similia in the treatment of artificial diseases produced by crude drugs. Will it cure it in every case? I might answer this question by saying yes and no. If the case is an uncomplicated one, or the history points to some suppression, it is almost certain to be the remedy. Again, you may first have to counteract psora or some other miasm, or *vice versa*, depending much on the nature of the symptoms, just as you do in treating the natural acute disease. Often, by counteracting the miasm present you cure the acute condition. The life-force will often throw off the effects of the drug when the psora is removed. I often think that the effects of any crude drug upon the organism is but the peculiar way in which it stirs up some latent miasm that has been smoldering in the organism; but in uncomplicated cases there is no truer simillimum than a high potency of the drug that produced the disease.

Drs. Hering, Lippe, and Swan had recognized this fact, but strange to say, had not recognized the law. Dr. H. C. Allen, as far back as 1880, recommended Rhus-tox.$^{200}$, which was then considered a high potency, for the cure of Rhus-tox. poisoning. Dr. Hering, in his *Guiding Symptoms*, refers us to the fact that Mercury or Opium if given in a high potency will often cure the effects of the crude poison. I am asked by many how long I usually wait after giving the high potency for antidotal purposes. My reply is, "Just as you do in the treatment of any chronic." I have cured acute cases of Rhus-tox. poisoning in three days, and have had to treat a chronic one a year before it was cured. Any remedy may be somewhat similar to the case and palliate (physiologically speaking), but the dynamitized poison is the highest phase of similia in the antidotal treatment of drug disease. It at once cancels its action upon the life force, so it can no longer prey upon the organism. Its forces are *spent;* it has met its *positive;* or, in other words, it has assisted the life forces in throwing off the disturbing force. We call this antidotal treatment, but the same thing might be said of the natural disease. We *are forever losing sight of the principle that Hahnemann discovered disease to be—the disturbed life force; and what difference does it make whether this life force be disturbed by the dynamitized powers of a psoric poison or by Quinine, by the tonic effects on the organism of some zymotic poison or the effects of Strychnine?* The *modus operandi* are the same. Can you remove the effects of the toxic poison, or entirely free the system from them, by aid of the stomach pump or by an emetic or cathartic? No. Can you purge them out? No—from the fact that any crude drugs, irrespective of quantity given or time the patient may be under its influence, may produce a drug disease. A single dose of Castor Oil or Castoria given to a delicate infant has been known to produce a persistent constipation or paresis of the bowels that required months to cure. The inconsistency of professing to remove or cure the bad effects of one crude drug by giving another—fully demonstrated in the Keely cure to remove or cure chronic alcoholism. Mechanically we are after the drug, and dynamically we are after its effects upon the life forces. The first often saves life, but it does not remove the cause; the disturbed life force has not been touched.

Iron for Arsenicum poison and Iodine or the Sulphate of Zinc for Rhus poisoning, appplied locally, is the science of a school of medicine that is only acquainted with the material man. It knows not Hahnemann's man, nor his laws that govern life. Their gods, though "dead, long dead," they worship still. Shall we, too, be hero worshipers, and not follow up the wonderful law of Hahnemann's?

No one with a reasoning mind can study a natural law (nor a spiritual one, for that matter), but that it will unfold itself to him in a clearer light and on a broader basis. The light comes to those who look for the light; and is not this so-called isopathy but similia upon a broader basis? They tell us if we use a high potency of a drug to cure or antidote the artificial disease that it has produced, we are practicing isopathy in place of Homœopathy. Well, if isopathy is a law of cure let us have it. The law of nature is the will of God, but I do not believe it is a natural law, nor do I think it exists in the material world. It is, therefore, a misnomer. Nature everywhere teaches us that there are no two things exactly alike. The same may be said of the forces of nature. The men who are seriously investigating and searching for the truth in this direction think otherwise. To them the star has made its appearance, and the truth must follow a careful and unprejudiced investigation. See *Organon*, 18, 28, 29. A similar disease cancels a similar disease. And since between idem and æquale can only be simillimum, it is, therefore, a similia of the highest degree of resemblance.

Hahnemann says: "Some would like to create a fourth mode of applying medicine in disease—by isopathy, so-called. But, granted this could be done," he says, "though it would be an inestimable invention"—it would still effect a cure by similia, since the miasm or drug, as the case may be, is given only highly potentized or in an altered condition.

How many times have you selected with all care and good judgment the antipsoric remedy for your patient, only to see failure follow its administration; whereby probably a score of previous cases had been cured or the result was all you had expected. Look carefully over your case again and you will find symptoms of cinchonism, mercurialism, nitrate of silver, the narcotics, patent medicines of some kind, or probably chronic alcoholism. Antidote any of the above that may be present; then, if your antipsoric remedy be still indicated, you will get results that will astonish you.

Do not think for a moment that I advocate the giving of a high potency of Quinine because the patient has taken Quinine, or of Mercury because he has taken Mercury, but because you find the symptoms of it in your patient. Even if some antipsoric remedy may seem to be better indicated in the case, antidote the natural disease first, as many times you will find the crude drug the exciting cause of stirring up psora, sycosis, or latent syphilis in your patient, on the removal of which the miasmatic disturbance subsides or disappears—a fact which I have tried to demonstrate by a number of cases given at the close of this article.

The more you investigate and study into this subject, the more you see that the so-called natural disease is but the outgrowth of an artificial one engrafted upon a psoric or miasmatic basis. Had the patient not suppressed an acute miasm by Quinine or endeavored to make a psoric liver act by toxic (misnomened physiological) doses of Mercury, he could have passed along through life very fairly and without much annoyance, so far as his chronic psora was concerned; and your so-named disease (pathological condition) would not have been the result.

Case I.—Mr. B., aged forty-eight years. Has suffered for years with chronic enlargement and hardening of the liver. There is a sensation of weight and heaviness in the liver, with an almost constant dull pain in the lower lobe. The stools are hard, dry, scanty, and clay-like. Never has stool unless artificial means are employed. Has been idle for a year past, and for past three months confined to his bed. During that time he has been gradually emaciating and losing strength. His taste is either metallic or entirely lost. He has no appetite, and mentally is despondent and discouraged, with no hope of recovery. He sweats easily and profusely at times. His circulation is very poor; nose, ears, knees, hands, and feet cold. The gums, teeth, tongue show marks of Mercury. He has taken it more or less all his life, and frequently in the six months previous to my taking the case. He says it is the only thing that helps him. Began treatment by giving two powders, Calomel$^{dmm}$ (Swan), and since that time four doses of the CM (Fincke). He has been under treatment but three months and is now almost well. Constipation has disappeared, stools are natural, appetite good; sleeps well and almost in any position. Previous to my treatment he could only sleep when lying on his back. He now works every day and is fully on the road to recovery.

Case II.—George L., aged forty. Has suffered with asthma for four years, and for the last two years has been unable to lie down. During the day he moves about with comparative effort, but at night is compelled to sleep in his chair, sitting almost erect. Family history good. Five years ago he suffered with third-day ague. Quinine was prescribed by three different physicians without relief, when he purchased a bottle of it, taking daily powerful doses of it for some time, of course suppressing the chills. Scarcely a year had passed until the asthma made its appearance, and a further knowledge of the case revealed no cause for his present trouble but the suppression of the chills by the over-doses of Quinine. Chinin-sulph.$^{cm}$, in water, was given, with directions to take a teaspoonful every two hours until five doses were taken. Results: Fourth day after taking, slight chill; asthma somewhat better. Seventh day, quite a severe chill. Was called to see him soon after the chill, but did not change the prescription. Tenth day, slept all night in bed very comfortably. On the fourteenth day he again reported that for the past few days he had slept all night except between the hours of twelve and one, during which time he was compelled to sit up, but again went to sleep and slept very comfortably until morning. His symptoms now are as follows: Mouth dry as a chip; tongue brown and crisp; lips covered with a whitish deposit like flour; thirst intense for small and frequent sips of cold water. He has stopped taking the medicine. He says, though it has helped his asthma, it is poisoning him, for he feels just as he did when taking an asthma specific one year ago, the principal constituent of which was Arsenic. His suffering from thirst was so great that I was induced to give him Arsenicum$^{cm}$, which has removed all his symptoms, and he has had no return of the asthma. I have never met exactly this latter group of symptoms except in the last stages of *diabetes mellitus*.

CASE III.—Mr. A. A. and wife, farmers, both strong and healthy, although they have a family history of psora. They came to me for treatment in September, 1891. Both were suffering from Rhus-tox. poisoning, contracted from picking berries in a low, marshy district, where this vine grew in abundance. These cases were the severest that I have ever witnessed. Their bodies were almost entirely covered with vesicles filled with a yellowish, watery fluid, which, when broken, ulcerated, and in many cases ran together, leaving large, raw, ulcerated surfaces. It was worse upon the extremities, head and neck. Rhus-tox.$^{cm}$ cured both of these cases apparently in one month, but it returned again exactly one year from that time, when I prescribed Sulphur$^{cm}$, based upon the psoric symptoms, followed by Rhus$^{50m}$. Mrs. A. was pregnant at the time, and when her child was born it had a very similar eruption all over the body, which was also cured by Rhus. A crop of boils followed some months later, showing that it had stirred up psora, which was cured by Hepar.

CASE IV.—Alice B., aged thirty. Erysipelas of the face eight years ago. Locally, Iodine had been applied. Since that time has had pain in left hip almost constantly. Was fleshy before erysipelas, but now is quite thin. Iodine$^{cm}$ reproduced the erysipelas eruption, which was cured by Rhus-tox. Pain left on the appearance of the eruption, never to return.

CASE V.—Osa S., aged sixteen. Light blonde; fleshy. Menstruated fifteenth year. One year after a goitre made its appearance, which was treated locally with Iodine tincture. A month later chorea made its appearance; cured with one prescription, Iodine$^{cm}$ (Fincke).

CASE VI.—Alice B.; constipation for twelve years. No relief but by enemas. History of case revealed that she was salivated previous to her trouble. Calomel$^{dmm}$ (Swan), one dose, cured.

